# Waterlogging in soil restricts the growth of *Gleditsia sinensis* seedlings and inhibits the accumulation of lignans and phenolic acids in thorns

**DOI:** 10.7717/peerj.17137

**Published:** 2024-03-22

**Authors:** Zai-Qi Luo, Xiao-Qian Shi, Xian-Ying Wang, Qiu-Lan Yang, Xin Pan, Wen-Xia Pan, Chun-Li Luo, Shan-Shan Yu, Wen-Wen Zhou, Bin-Rui Ren, Yin Yi, Xi-Min Zhang

**Affiliations:** 1 Key Laboratory of State Forestry Administration on Biodiversity Conservation in Karst Area of Southwest, Guizhou Normal University, Guiyang, China; 2 Guizhou Academy of Forestry, Guiyang, China; 3 School of Life Sciences, Guizhou Normal University, Guiyang, Guizhou, China; 4 Key Laboratory of Environment Friendly Management on Alpine Rhododendron Diseases and Pests of Institutions of Higher Learning in Guizhou Province, Guizhou Normal University, Guiyang, China

**Keywords:** *Gleditsia sinensis*, Water-holding capacity, Soil water potential, Secondary metabolites, UHPLC-MS

## Abstract

*Gleditsia sinensis*, commonly known as Chinese Zaojiao, has important economic value and medicinal compounds in its fruits and thorns, making it widely cultivated artificially in China. However, the available literature on the impact of waterlogging on the growth of *G. sinensis* seedlings and the accumulation of metabolite compounds in its thorns is limited. To address this knowledge gap, *G. sinensis* seedlings were planted in soil supplemented with pindstrup substrate, which enhances the water-holding capacity of the soil. The analyses of morphological traits and nutrient elements in one-year-old *G. sinensis* seedlings grown naturally under ambient conditions and metabolite accumulation in its thorns were conducted. The results showed that the waterlogged soil significantly diminished the height, fresh weight, and dry weight of seedling roots and stems (*P* < 0.05). Furthermore, waterlogging hindered the uptake of iron (Fe) and manganese (Mn), as well as the transport of potassium (K). The identified metabolites within the thorns were categorized into 16 distinct groups. Relative to the control soil, fatty acids and derivatives were the most down-regulated metabolites in the waterlogged soil, accounting for 40.58% of the total metabolites, followed by lignans (38.71%), phenolic acids (34.48%), saccharides and alcohols (34.15%), steroids (16.67%), alkaloids (12.24%), flavonoids (9.28%), and glycerophospholipids (7.41%). Conversely, nucleotides and derivatives experienced the greatest up-regulation in the waterlogged soil, accounting for 50.00% of the total metabolites. In conclusion, waterlogging negatively impacted the growth of *G. sinensis* seedlings and inhibited the accumulation of metabolites. Hence, when considering the accumulation of secondary metabolites such as lignans and phenolic acids, appropriate management of soil moisture levels should be taken into account.

## Introduction

The genus *Gleditsia* Linn. encompasses a diverse array of plants that are classified under the Leguminosae family. These plants boast a global distribution, being found in various regions across Asia, tropical Africa, and the Americas. There are approximately 16 documented species worldwide. Within China, specifically, the genus comprises six distinct species along with two varieties (http://www.iplant.cn/info/Gleditsia?t=foc). These species are primarily distributed across several provinces, including Hebei, Shanxi, Fujian, Guangdong, Guangxi, Shanxi, Ningxia, Gansu, Sichuan, Guizhou, Yunnan, Shandong, Jiangsu, and Zhejiang (http://www.iplant.cn/info/Gleditsia%20sinensis?t=foc). Among the Gleditsia genus, *Gleditsia sinensis* stands out as a prominent species commonly referred to as Chinese Zaojiao.

*Gleditsia sinensis*, due to its considerable economic value, offers various versatile applications. Its pod yields a juice that serves as an effective substitute for soap in the washing of silk and wool fabrics. Its young shoots, when combined with oil and salt, can be utilized as a culinary ingredient. Additionally, the cooked and candied endosperm of seeds can be consumed directly. Moreover, the dried thorns of *G. sinensis* contain a diverse array of potent medicinal compounds and exert inhibitory effects on the proliferation of colon cancer cells, liver cancer cells, gastric cancer cells, and smooth muscle cells (Lee et al., 2010; Yu et al., 2015; Lee et al., 2013; Lee et al., 2012). Consequently, its thorns have gained widespread usage as an herbal medicine in various Asian countries. In traditional Chinese medicine, it is frequently prescribed as an adjunctive therapy for the treatment of breast cancer, lung cancer, and other types of cancer (Zhang et al., 2016).

Despite its significant economic value, the *G. sinensis* tree remains relatively scarce, primarily observed in sparsely distributed regions near residential areas. In recent years, the province of Guizhou has initiated efforts to vigorously develop characteristic forestry in support of rural revitalization. For example, in 2021, the cultivation of *G. sinensis* within this province is projected to yield a newly planted area of 20.37 thousand hectares, contributing to a total area of 893 thousand hectares (http://www.forestry.gov.cn/main/5384/20210724/172550581923033.html). Currently, it is known that the species is a deciduous tree that prefers abundant sunlight and has robust tolerance to drought conditions. Remarkably, *G. sinensis* demonstrates adaptability to various soil types, including limestone soil, acidic soil, clay soil, sandy soil, and even slightly saline-alkaline soil (Geyer, 1993; Tilstone et al., 1998). Therefore, understanding the plasticity of this species in facing different abiotic conditions present in the soil can directly contribute to the forestry of this species.

Soil moisture stands as a critical environmental parameter that significantly impacts the normal growth and survival of plants (Liang et al., 2019). In recent years, due to climate change, the global hydrological cycle has undergone alterations, resulting in uneven patterns of rainfall intensity and distribution. Heavy rainfall frequently leads to the saturation of soil moisture, particularly in areas with low-lying terrains, clay-rich soils, or insufficient drainage conditions, ultimately causing a waterlogging stress (Herzog et al., 2016; Pan et al., 2021). Waterlogging can impede the entry of oxygen into the root system, resulting in hypoxia and root suffocation, which can have adverse effects on plant growth and development. Previous studies have examined the relationships between various environmental factors, such as light exposure, exogenous calcium levels, and drought stress, and the growth of *G. sinensis* (Liu et al., 2023; Guo et al., 2021; Yan et al., 2006). Nevertheless, there is a paucity of research focusing on the impact of waterlogging on the growth of *G. sinensis* seedlings. Consequently, it is necessary to further study the potential effects of waterlogging on the growth of *G. sinensis* seedlings.

To address this objective, one-year-old *G. sinensis* seedlings were used for a study to investigate the growth patterns and metabolite accumulation in response to excessive water levels in the soil. The main objective of this study was to examine the impact of waterlogging on the growth of *G. sinensis* seedlings and the accumulation of metabolites in its thorns. To achieve this, we tested three hypotheses: (1) waterlogging inhibits the growth of seedlings, including their root system and plant height; (2) waterlogging affects the absorption and transportation of certain nutrient elements; (3) waterlogging hinders the accumulation of secondary metabolites in its thorns. By doing so, this research endeavors to shed light on the complex relationship between waterlogging, *G. sinensis* growth dynamics, and the synthesis of key metabolites.

## Materials & Methods

### Soil physical and chemical properties

The soil matrix used in this study was divided into two distinct treatment groups: control soil and pindstrup soil. The control soil consisted of a blend of garden soil (purchased from the flower market in Guiyang city) and humus soil (purchased from Organic Fertilizer Development Co., Ltd. in Guiyang city) in a 1:1 (v/v) ratio. The pindstrup soil comprised a mixture of garden soil, humus soil, and Pindstrup moss peat substrate (sourced from Pindstrup Company, Ryomgaard (Kongerslev), Denmark) in a ratio of 0.5:0.5:1 (v/v). Under natural conditions, the pindstrup substrate possesses notable water retention capabilities. To facilitate the experiment, the mixture of soils was then placed in black tree planting bags (25 cm in diameter, 20 cm in height) with free drainage at the bottom. Both types of soil were adequately irrigated, and the soil moisture within the planting bags was monitored within a controlled greenhouse environment. During the measurement process, soil samples were extracted from a depth of 10 cm, with four points sampled and meticulously blended within each planting bag to form one replicate. The soil water potential was promptly determined using a dew point potential meter (WP4-T). Subsequent to the measurement, the fresh soil samples were immediately weighed, dried at 60 °C to a constant weight, and weighed again to determine the dry weight. The soil water content was calculated using the formula: soil water content = (fresh weight−dry weight)/dry weight × 100%. Each treatment was subject to four replicates of soil measurements to ensure statistical robustness.

To examine the disparities in nutrient elements between the two soil types, the nutrient content within each soil was determined. For further analysis, the samples (through a 100-mesh sieve) were digested using microwave-assisted techniques, and the metal element content (such as K, Ca, Mg, Fe, Cu, Zn, Mo, and Mn) was measured using Inductively Coupled Plasma-Mass Spectrometry (ICP-MS) following appropriate filtration (Yu et al., 2014). The quantification of metal elements was carried out by referencing the standard curve obtained from the analysis of a mixed standard sample of metals (GNM-M1810581-2013). The total nitrogen (TN) content of the soil was determined through the H_2_SO_4_–K_2_Cr_2_O_7_ method (Bao, 2020). The total phosphorus (TP) content of the soil was also ascertained employing the molybdenum blue method (Liu et al., 2020).

### Seed germination and seedling growth

Mature pods of *G. sinensis* were meticulously collected from Quanhu Park in Guiyang city, located in Guizhou Province (106°62′E, 26°67′N) and placed in a storage cabinet. Prior to planting, the seeds were subjected to a preparatory treatment. Firstly, they were soaked in concentrated sulfuric acid for duration of 2 h to soften the seed coat (Liu et al., 2023), followed by thorough rinsing with tap water on three separate occasions. Subsequently, the seeds were immersed in tap water at room temperature until they achieved full hydration and experienced notable swelling. The swollen seeds were then planted in the aforementioned tree planting bags, allowing them to grow naturally under ambient conditions (natural rainfall) for a period of one year before commencing with various measurements.

### Measurement of seedlings morphological traits

The seedlings were carefully extracted from the planting bags, and the rhizosphere soil was thoroughly rinsed with tap water. Next, the roots were precisely severed at the junction of the roots and stems, and the excess water on the roots was carefully blotted using filter paper. The fresh weight of the roots was recorded, after which the roots were dried at 60 °C to a constant weight, facilitating the determination of the dry weight of the roots. From each seedling, a random selection of four compound leaves was made, and the number of leaflets was accurately recorded. Subsequently, the average number of leaflets was calculated. Additionally, the length of five thorns, located approximately 10 cm from the stem, was meticulously measured from a representative seedling, enabling the determination of the average length of thorns. All the leaves of a single seedling were excised, and the fresh weight of the leaves was measured. Following this, the leaves were dried at 60 °C to a constant weight, facilitating the determination of the dry weight. Furthermore, the lateral branches were pruned from a seedling, and the stem was cut off. The fresh weight of the stem was measured. Subsequently, the stem was dried at 60 °C to a constant weight, allowing for the determination of the dry weight. Four seedlings were used as biological replicates for each treatment.

### Determination of nutrient elements in plant tissue

The previously dried plant roots, stems, and leaves were subjected to further processing for the purpose of determining the content of nutrient elements. K, Ca, Mg, Fe, Cu, Zn, Mo, and Mn were quantified using inductively coupled plasma-mass spectrometry (ICP-MS) according to the method of Yu et al., (2014).

### Metabolite detection

All of the thorns derived from the stems of a single seedling were meticulously collected. Each experimental treatment comprised 6 seedlings, ensuring an adequate number of biological replicates. Subsequent to thorn collection, they were promptly transferred to centrifuge tubes and rapidly frozen using liquid nitrogen. The frozen thorns were then stored at −80 °C. Approximately 20 mg of the thorn samples were accurately weighed. Subsequently, 1,000 µL of an extraction solution (methanol:acetonitrile:water = 2:2:1(v/v)) containing internal standard mixture was added to each sample. The samples were subjected to grinding at a frequency of 35 Hz for duration of 4 min, followed by sonication for 5 min in an ice bath, with the process repeated three times. After the samples were left at −40 °C for 1 h, they were centrifuged at 12,000 rpm at 4 °C for 15 min. The supernatant was taken and used for detection. An equal amount of the supernatant was mixed to create a quality control (QC) sample, which was used to analyze the repeatability under the same processing method.

The compounds were separated utilizing an Ultra High Performance Liquid Chromatography (UHPLC) (Vanquish; Thermo Fisher Scientific, Waltham, MA, USA) with a Phenomenex Kinetex C18 column (2.1 mm × 50 mm, 2.6 µm). The mobile phase consisted of two components: mobile phase A was a 0.1% acetic acid solution, and mobile phase B was acetonitrile:isopropanol (1:1,v/v). A gradient elution method was employed according to the following time intervals: 0–0.5 min, 1% B; 0.5–4 min, 1%–99% B; 4–4.5 min, 99% B; 4.5–4.55 min, 99%–1% B; 4.55-6 min, 1% B. The column temperature was 25 °C, the injection chamber temperature was 4 °C, the flow rate was 0.3 mL/min, and the injection volume was 2 µL.

Mass Spectrometry (MS) (Orbitrap Exploris 120; Thermo Fisher Scientific, Waltham, MA, USA) combined with control software (Xcalibur, version 4.4; Thermo Fisher Scientific, Waltham, MA, USA) was employed to facilitate the acquisition of both primary and secondary mass spectrometry data. The detailed parameters were as follows: sheath gas flow rate (50 Arb), aux gas flow rate (15 Arb), capillary temperature (320 °C), full MS resolution (60000), MS/MS resolution (15000), collision energy (SNCE 20/30/40), spray voltage (3.8 kV (positive) or −3.4 kV (negative)).

### Metabolite identification and data processing

The raw data was processed and converted into mzXML format using the ProteoWizard software. Peak detection and annotation were accomplished through the utilization of an in-house R program and the BiotreeDB database (version 2.1). For multivariate analyses, including orthogonal projections to latent structures-discriminant analysis (OPLS-DA) and principal component analysis (PCA), the MetaboAnalyst 5.0 was employed. In the OPLS-DA analysis, the variable importance in the projection (VIP) value of the first principal component was obtained. Metabolites with a VIP >1 and a *P*-value < 0.05 (Student’s *t*-test) were considered to be significantly altered metabolites between the groups. Following the identification of differential metabolites, a subsequent Kyoto Encyclopedia of Genes and Genomes (KEGG) enrichment analysis was conducted on the differential metabolites.

### Statistical analysis

The data were presented as mean ± standard deviation (SD) and assessed for statistical significance using independent sample *t*-tests conducted in SPSS version 18 (*P* < 0.05).

## Results

### Pindstrup substrate maintained waterlogging in soil

**Figure 1:**
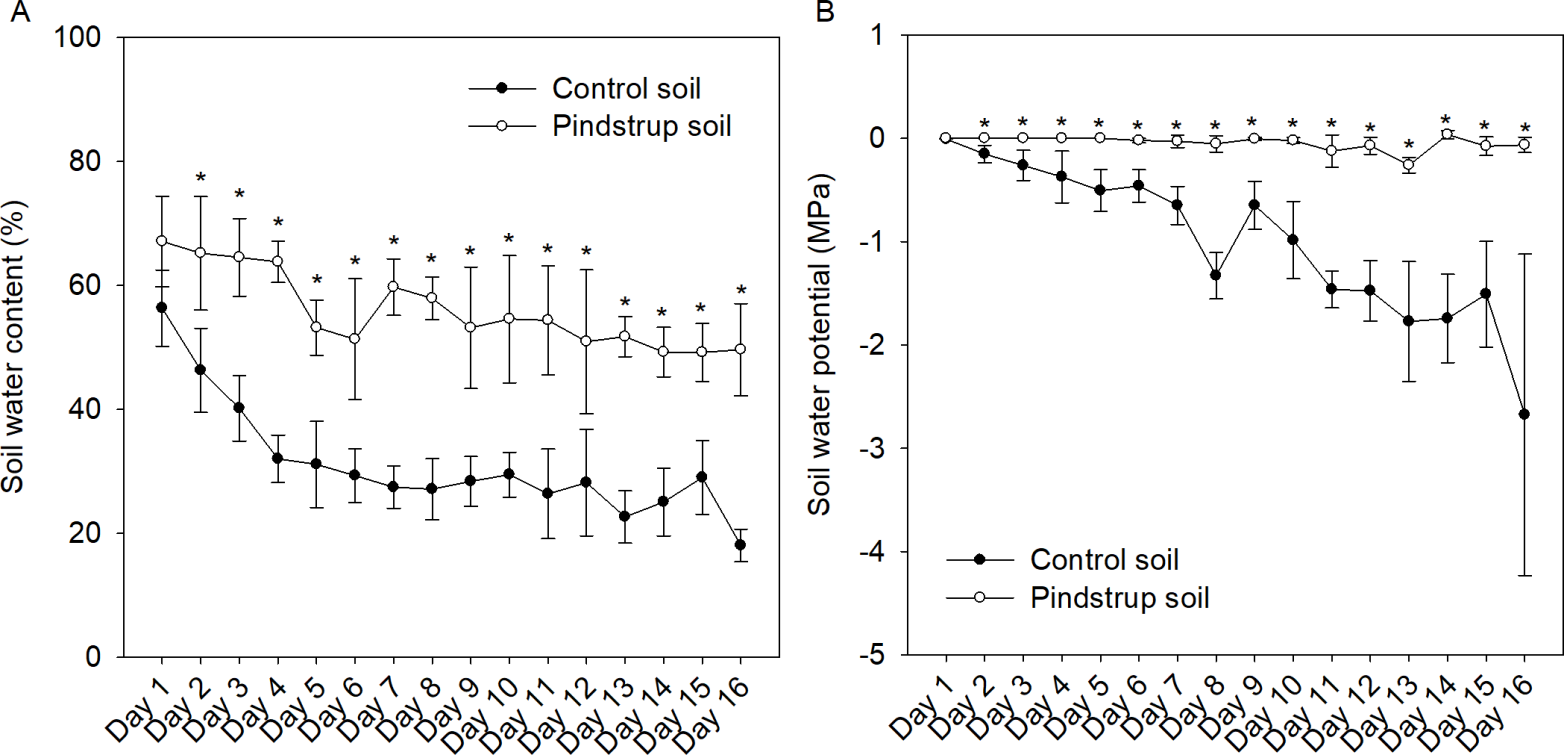
Soil water content (A) and soil water potential (B) in control soil and pindstrup soil. The data represent the mean ± SD (*n* = 4). Statistical analysis was performed using *t*-tests and asterisks represent significant differences between control soil and pindstrup soil in the same day (*P* < 0.05).

The water content and water potential of both soil types were continuously monitored for a period of 16 days (once a day). The findings revealed a decreasing trend in relative water content for both the control soil and the pindstrup soil. However, at a specific monitoring time point (*e.g.*, the 9th day), the water content of the pindstrup soil (53.14%) was significantly higher than that of the control soil (28.36%) (Fig. 1A). In contrast to the results observed for soil water content, the water potential of the pindstrup soil did not display significant changes throughout the 16-day measurement period. Notably, the water potential in the pindstrup soil was found to be significantly higher than that in the control soil (Fig. 1B). These results suggest that the pindstrup soil is capable of maintaining a higher level of available water in the soil matrix, even after a prolonged period without regular irrigation.

### Inhibition of seedling growth due to waterlogging

**Figure 2:**
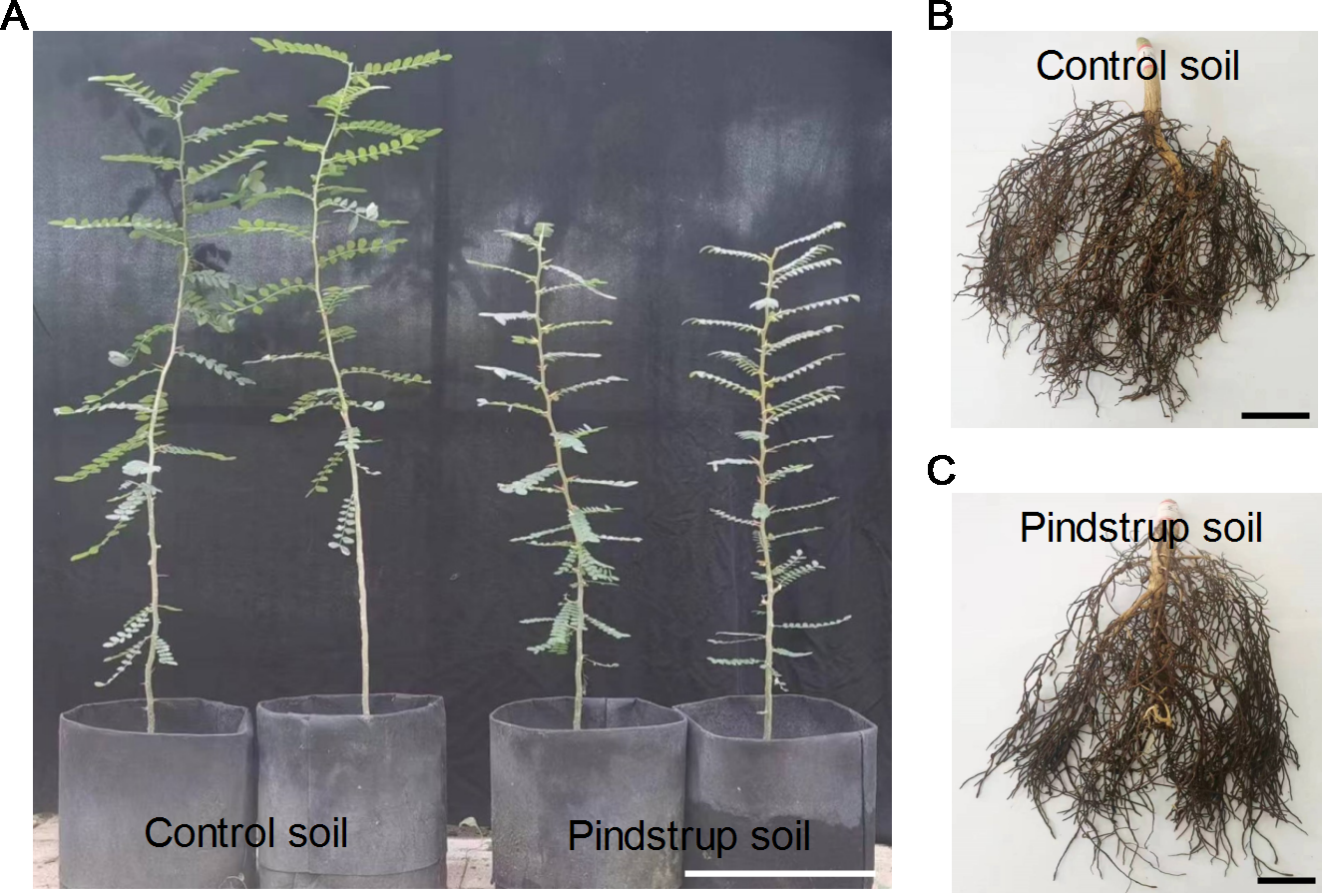
The growth phenotypes of *G. sinensis*. seedlings grown in control soil and pindstrup soil. (A) Show the height of one-year *G. sinensis* seedlings growing in two types of soil. Bar = 20 cm. (B) and (C) show the root growth of one-year *G. sinensis* seedlings growing in control soil and pindstrup soil, respectively. Bar = 2 cm.

After one year of growth, some morphological traits of the seedlings were measured in both soil types. The results revealed significant differences in plant height, stem fresh weight, root fresh weight, stem dry weight, and root dry weight between the control soil (with measurements of 105.53 cm, 27.63 g, 19.65 g, 10.95 g, and 6.27 g, respectively) and the pindstrup soil (with measurements of 70.66 cm, 13.73 g, 12.10 g, 5.48 g, and 3.38 g, respectively) (*P* < 0.05) (Fig. 2; Table 1).

**Table 1:** Morphological traits of seedlings grown in control soil and pindstrup soil. The data represent the mean ± SD (*n* = 4). Statistical analysis was performed using *t*-tests and different lowercase letters in the same line represent significant differences between control soil and pindstrup soil (*P* < 0.05).

Morphological traits	Control soil	Pindstrup soil
Plant height (cm)	105.53 ± 13.52a	70.66 ± 10.87b
Thorn length (cm)	2.36 ± 0.24a	1.98 ± 0.44a
Leaflet number (N)	13.75 ± 2.87a	13.50 ± 2.38a
Stem diameter (cm)	0.71 ± 0.12a	0.64 ± 0.08a
Leaf fresh weight (g)	14.98 ± 3.11a	10.07 ± 2.85a
Stem fresh weight (g)	27.63 ± 8.34a	13.73 ± 5.06b
Root fresh weight (g)	19.65 ± 4.98a	12.10 ± 2.88b
Leaf dry weight (g)	4.86 ± 1.09a	3.22 ± 0.89a
Stem dry weight (g)	10.95 ± 3.60a	5.48 ± 1.92b
Root dry weight (g)	6.27 ± 1.74a	3.38 ± 1.04b

### Influence of waterlogging on the nutrient content of seedlings

**Figure 3:**
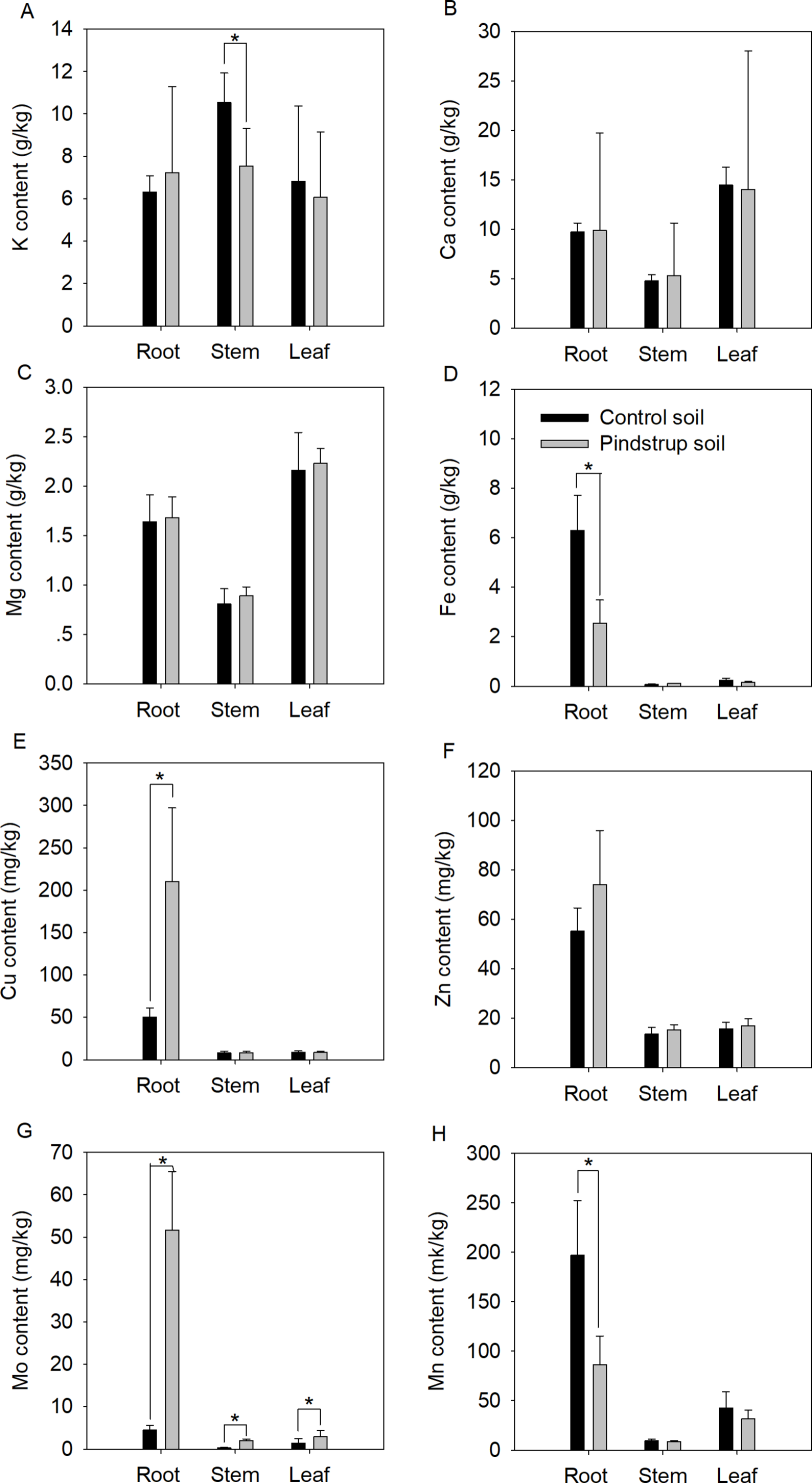
K content (A), Ca content (B), Mg content (C), Fe content (D), Cu content (E), Zn content (F), Mo content (G), Mn content (H) of root, stem and leaf in one-year *G. sinensis* seedlings growing in control soil and pindstrup soil. The data represent the mean ± SD (*n* = 4). Statistical analysis was performed using t-tests and asterisks represent significant differences between control soil and pindstrup soil (*P* < 0.05).

Nutrient elements of the two soils were analyzed to determine whether there was a significant difference between them. The results showed a significantly higher Ca content in the pindstrup soil (31.14 g kg^−1^) compared to the control soil (13.61 g kg^−1^). However, no significant differences were observed in the measured nutrient elements content, including TN, TP, K, Mg, Fe, Cu, Zn, Mo, and Mn (Table S1). To further investigate potential differences in nutrient elements in *G. sinensis* seedlings grown in the two soil types, the contents of nutrient elements in the roots, stems, and leaves of one-year-old seedlings were measured. The results indicated that the contents of Fe and Mn in the roots of seedlings cultivated in the control soil were significantly higher compared to those in the pindstrup soil. Conversely, the contents of Cu and Mo in the roots of seedlings grown in the control soil were significantly lower than those in the pindstrup soil (*P* < 0.05) (Fig. 3). In the stems of seedlings grown in the control soil, the K content was significantly higher than that in the pindstrup soil, while the Mo content was significantly lower (*P* < 0.05) (Fig. 3). Furthermore, the Mo content in the leaves of seedlings grown in the control soil was significantly lower than that in the pindstrup soil (*P* < 0.05) (Fig. 3); however, no significant differences were found in the contents of other elements.

### Screening and analysis of differentially abundant metabolites in *G. sinensis* thorn

**Figure 4:**
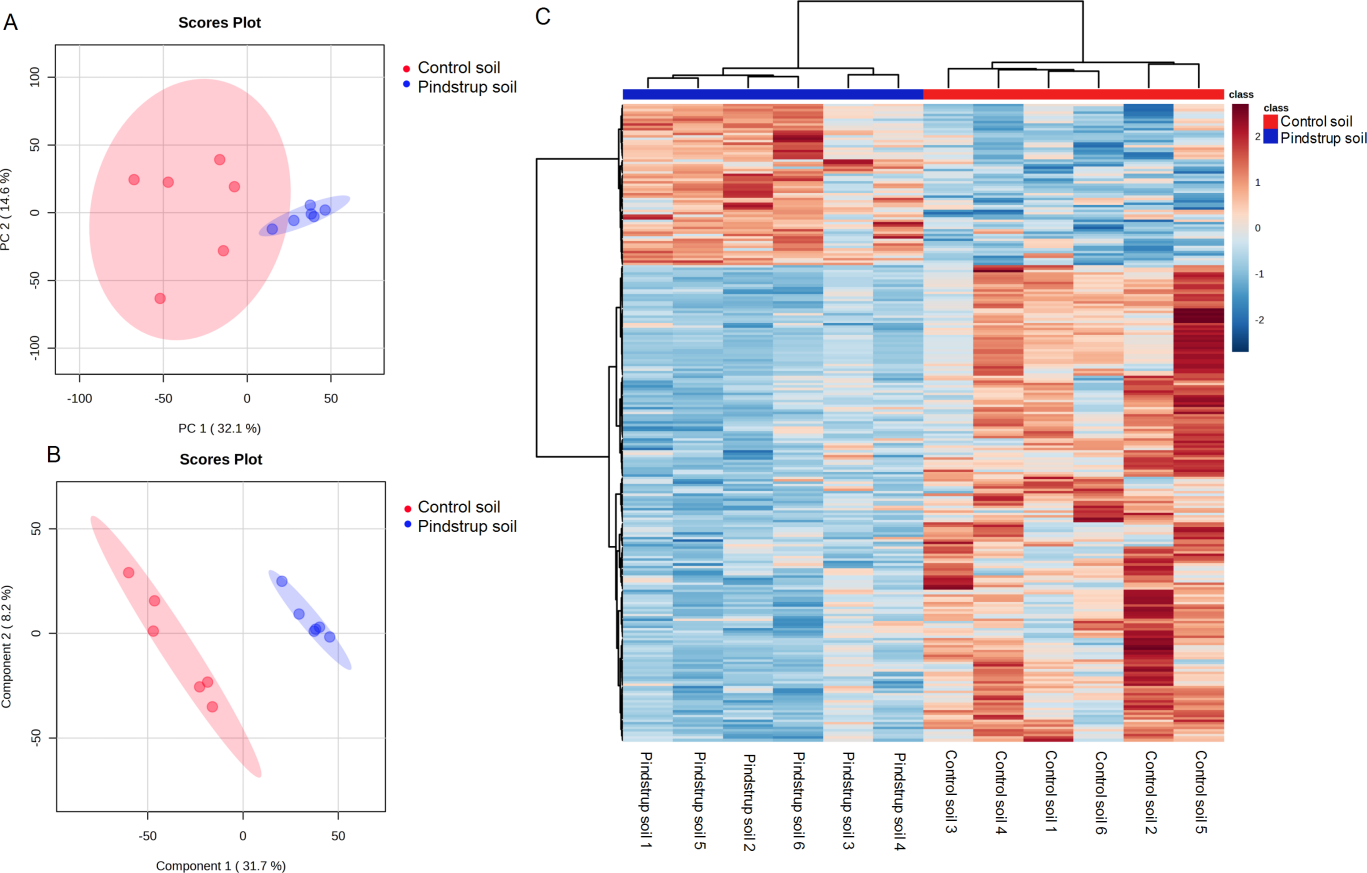
Metabolite identification of thorns in *G. sinensis*. (A) PCA of samples with six biological repetitions. (B) OPLS-DA of samples with six biological repetitions. The red and blue circles display 95% confidence regions of control soil and pindstrup soil. (C) Hierarchical clustering analysis of the identified metabolites from thorns of *G. sinensis* grown in control soil and pindstrup soil. Columns and rows represent individual metabolites and different samples, respectively.

Using the UHPLC- MS detection platform, total ion chromatograms (TIC) of thorn samples were obtained under both positive and negative ion modes for the two treatments (Fig. S1). The total ion flow of thorn samples was comparable between the two treatments, but differences in peak area were observed. By matching with the mass spectrometry database (Biotree DB V2.1), a total of 836 metabolites were annotated (Table S2). PCA was performed on all detected metabolites, including quality control samples, to elucidate the variations both between and within treatment groups. The PCA analysis revealed that PC1 and PC2 accounted for 32.1% and 14.6% of the total variation, respectively (Fig. 4A). To obtain more reliable differences, OPLS-DA was utilized to filter the orthogonal variables in metabolites. The OPLS-DA successfully distinguished the metabolites of thorn in the control soil from those in the pindstrup soil (Fig. 4B). These findings indicate significant differences in the metabolites of thorn grown in the two different soil types.

To further explore the disparities in metabolites between the thorn samples from the control soil and pindstrup soil, differentially abundant metabolites were identified. A total of 265 differentially abundant metabolites (VIP >1 and a *P*-value <0.05) were screened between the thorn of pindstrup soil and control soil, and visualized through a heat map (Fig. 4C). Compared to the control soil, 67 metabolites were up-regulated, while 198 metabolites were down-regulated in the thorn of pindstrup soil (Fig. 4C, Table S3).

To investigate the impact of waterlogging on metabolite accumulation, the detected metabolites were classified into 16 categories (Table 2). Among the down-regulated metabolites, the most abundant category was Fatty acids and derivatives, accounting for 40.58%, followed by lignans (38.71%), phenolic acids (34.48%), saccharides and alcohols (34.15%), steroids (16.67%), alkaloids (12.24%), flavonoids (9.28%), and glycerophospholipids (7.41%) (Table 2). Among the up-regulated metabolites, the most abundant category was nucleotides and derivatives, accounting for 50.00%, followed by glycerophospholipids (18.52%) and steroids (13.89%) (Table 2). Overall, these results also indicated a more pronounced inhibiting effect of waterlogging on the primary metabolites of *G. sinensis* thorn.

**Table 2:** Differentially abundant metabolites found in thorns of *G. sinensis* grown in pindstrup soil in comparison with control soil. PM indicates primary metabolites; SM indicates secondary metabolites.

Primary classification	Type	Number of identified metabolites	Number of down-regulated metabolites	Ratio	Number of up-regulated metabolites	Ratio
Amino acids and derivatives	PM	49	12	24.49%	3	6.12%
Nucleotides and derivatives	PM	10	2	20.00%	5	50.00%
Organic acids and derivatives	PM	82	22	26.83%	7	8.54%
Steroids	PM	36	6	16.67%	5	13.89%
Saccharides and alcohols	PM	41	14	34.15%	3	7.32%
Glycerophospholipids	PM	54	4	7.41%	10	18.52%
Fatty acids and derivatives	PM	69	28	40.58%	4	5.80%
Alkaloids	SM	49	6	12.24%	2	4.08%
Coumarins	SM	36	8	22.22%	1	2.78%
Flavonoids	SM	97	9	9.28%	4	4.12%
Isoflavonoids	SM	15	4	26.67%	2	13.33%
Lignans	SM	31	12	38.71%	0	0.00%
Phenolic acids	SM	87	30	34.48%	3	3.45%
Tannins	SM	9	2	22.22%	0	0.00%
Terpenoids	SM	74	14	18.92%	8	10.81%
Others	SM	97	25	25.77%	10	10.31%
Total		836	198		67	

### Enrichment analysis of differentially abundant metabolites

**Figure 5:**
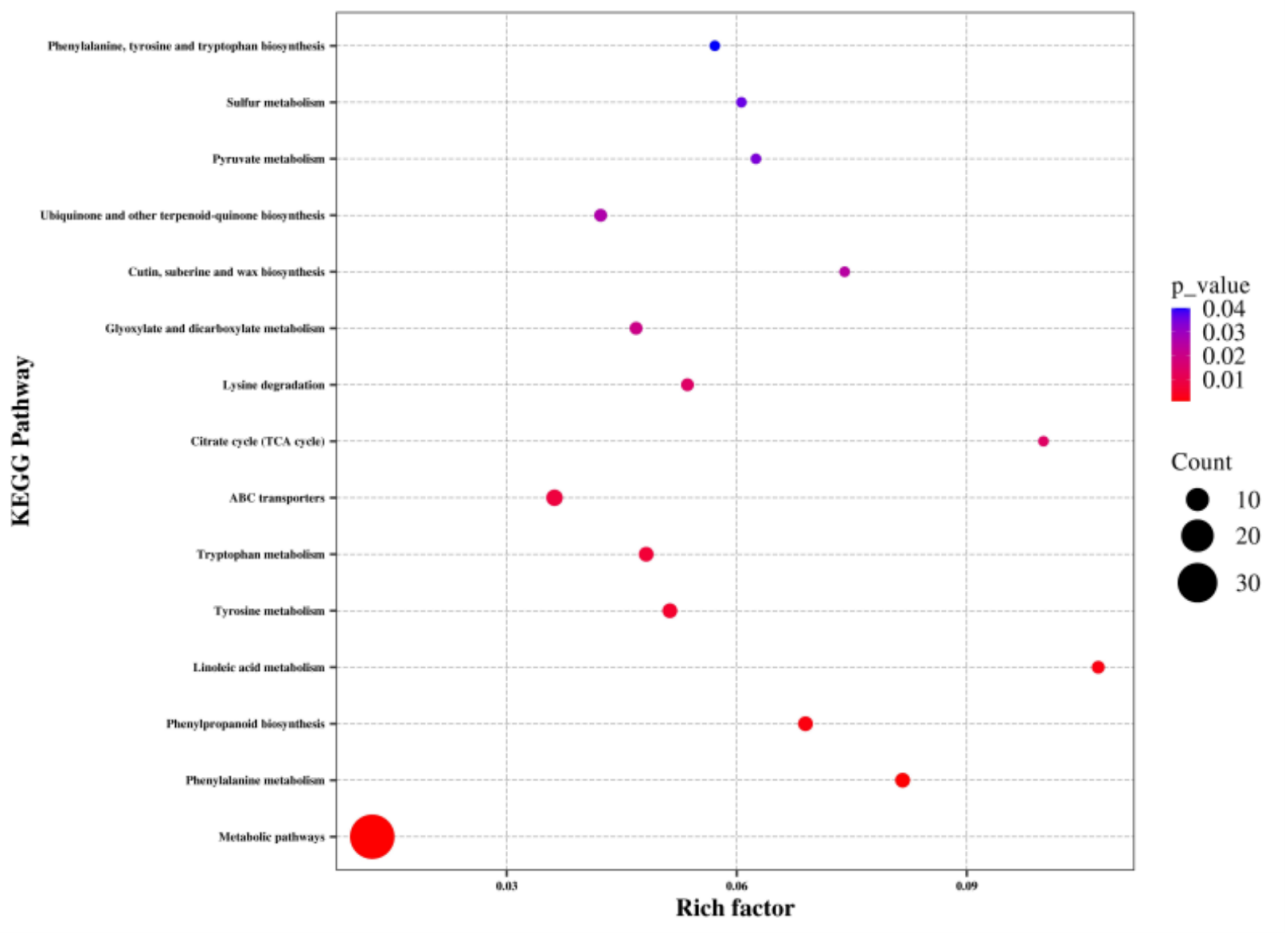
KEGG pathway analysis of differentially abundant metabolites of the thorns in *G. sinensis* based on pathway-matched metabolites.

To identify the key metabolic pathways affected by waterlogging, the differentially abundant metabolites were matched to the KEGG database for enrichment analysis. Overall, these metabolites were found to be involved in 66 KEGG pathways, including metabolic pathways and biosynthesis of secondary metabolites (Table S4). Among these pathways, metabolic pathways, phenylalanine metabolism, phenylpropanoid biosynthesis, linoleic acid metabolism, tyrosine metabolism, tryptophan metabolism, ABC transporters, citrate cycle, lysine degradation, glyoxylate and dicarboxylate metabolism, cutin, suberine and wax biosynthesis, ubiquinone and other terpenoid-quinone biosynthesis, pyruvate metabolism, sulfur metabolism, and phenylalanine, tyrosine, and tryptophan biosynthesis showed significant enrichment (*P* < 0.05) (Fig. 5). Notably, a significant number of the identified metabolic pathways were associated with amino acid biosynthesis and metabolism, indicating a substantial impact of waterlogging on these pathways.

## Discussion

Roots serve as the primary interface between plants and the soil, making them highly susceptible to water stress (Geng et al., 2018). Our findings showed that the pindstrup soil had significantly higher water content and soil water potential compared to the control soil after a two-week non-irrigation period (Fig. 1). These results suggested that pindstrup soil has high water retention capacity and will accumulate more water in the rhizosphere (Song et al., 2016; Carminati & Vetterlein, 2012), resulting in waterlogged stress for the *G. sinensis* seedlings during the growth stage. As our results demonstrate, in the pindstrup soil (excessive moisture), the fresh weight and dry weight of *G. sinensis* seedling roots were significantly lower compared to those in the control soil (Fig. 2 and Table 1), indicating inhibited root growth (Herzog et al., 2016). This inhibition may be due to waterlogging limiting the availability of rhizosphere O_2_ and excessive moisture damage to the roots (Herzog et al., 2016; Malik et al., 2002). In fact, certain drought-tolerant or waterlogging-sensitive species, such as *Quercus robur, Liquidambar styraciflua*, and *Lupinus angustifolius* (Kreuzwieser & Rennenberg, 2015; Schmull & Thomas, 2000; Neatrour, Jones & Golladay, 2007; Bramley et al., 2011; Colin-Belgrand, Dreyer & Biron, 1991), also experience inhibited root growth due to waterlogging. This was consistent with our results, suggesting that *G. sinensis* is a potential drought-tolerant species (Liu et al., 2020). Additionally, *G. sinensis* seedlings in the pindstrup soil showed a decrease in thorn length, leaflet number, stem diameter, leaf fresh weight, and leaf dry weight, especially significantly reduced plant height, stem fresh weight, and stem dry weight (Fig. 2 and Table 1). This is explained by the limitation of root biomass reduction caused by waterlogging, which has a significant impact on plant growth patterns (Schmull & Thomas, 2000). The observed growth phenotypes of the seedlings corroborated the morphological data. The integration of soil physical and chemical properties data demonstrated the inhibitory effects of waterlogging on the growth of *G. sinensis* seedlings.

Waterlogged trees often experience impaired nutrient absorption and transportation from roots to stems (Schmull & Thomas, 2000; Merchant et al., 2010). Our research results indicated that levels of Fe and Mn in *G. sinensis* roots exposed to waterlogging soil were significantly reduced (Fig. 3). This are attributed to root hypoxia caused by waterlogging, which hinders the entry of Fe and Mn into the roots (Jiménez et al., 2021), as well as reduced activities of proton ATPase and ferric chelate reductase (FCR) due to waterlogging, leading to decreased Fe and Mn uptake (Martínez-Cuenca et al., 2015). In contrast, Cu and Mo levels increased in roots grown in waterlogged soil, which are explained by enhancing Cu absorption and contributing to increased superoxide dismutase (SOD) activity to maintain the balance of reactive oxygen species within the plant (Chen, Chen & Yeh, 2011). Additionally, Mo uptake may activate root defense mechanisms to counteract damage caused by waterlogging stress on the roots (Hayyawi et al., 2020; Heshmat et al., 2021). K levels in stems were significantly lower than in control soil, indicating that waterlogging had the greatest impact on inhibiting K transport from roots to stems. In Pindstrup soil, Mo levels increased in stems and leaves, likely due to significant uptake and accumulation of Mo by the roots (Fig. 3G). Although significantly higher levels of Ca were present in the pindstrup soil, there was no significant difference in Ca levels in seedling tissues grown in both soils. It is speculated that this Ca content might not impact the growth of *G. sinensis* seedlings, as in Karst areas where *G. sinensis* trees thrive, the total Ca content in the soil can reach as high as 40.05 g kg^−1^ (Yang et al., 2021).

Recent scientific literature has witnessed significant contributions regarding the metabolic composition, isolation, and identification from the thorns of *G. sinensis* (Zhang et al., 2020; Zeng et al., 2023; Yu et al., 2017), the differences in chemical composition between the epidermis, xylem, and pith in thorns (Ya et al., 2022), metabolite accumulation in different tissues of *G. sinensis* under conditions of drought stress and rehydration (Liu et al., 2020). In our current study, a comprehensive analysis identified a total of 836 putative metabolites in the thorn (Table S2). Fatty acids play essential roles as precursors of cuticular waxes in the plant cuticle and are specific to various membrane lipids, contributing to membrane homeostasis. It has been reported by Ya et al. (2022) that fatty acids accounted for a considerable proportion of the thorn metabolites and may contribute to its hardness (Ya et al., 2022). However, in waterlogging soil, the levels of fatty acids and their derivatives were down-regulated, suggesting that this may affect the synthesis of cuticular waxes in *G. sinensis* thorn, thereby reducing their hardness. Lignans, secondary metabolites derived from phenylpropanoids, have been recognized for their bioactive properties in various human diseases, including colon and breast cancer (Záleák, Bon & Pospíil, 2019). Additionally, phenolic acids, another group of secondary metabolites, have shown potential as anticancer compounds, with many acting as cytotoxic agents that promote apoptosis, inhibit proliferation, and target cancer cells (Abotaleb et al., 2020). Importantly, our findings showed that waterlogging soil significantly decreased the accumulation of lignans and phenolic acids. There results suggest that soil moisture levels should be considered when utilizing these metabolites for future disease treatments.

There are variations in carbohydrate accumulation among different species. Jaeger et al. (2009) reported a significant decrease in carbohydrate content in waterlogging-sensitive plants. Consistent with these findings, our results indicate that carbohydrate accumulation was down-regulated in waterlogging soil (Table 2), indicating that *G. sinensis* may be a waterlogging-sensitive species from a carbohydrate perspective. Our results observed the accumulation of nucleotides and their derivative metabolites in waterlogging soil (Table 2). This is explained by the the decrease in amino acid content produced by glycolysis due to waterlogging, resulting in a decrease in the number of amino acids entering the tricarboxylic acid (TCA) cycle intermediates (Kreuzwieser et al., 2009) and nicotinamide adenine dinucleotide phosphate (NADP) consumption. Nucleotide substances need to be resynthesized to maintain the nucleotide pool homeostasis, thereby replenishing energy deficiency (Kreuzwieser & Rennenberg, 2015; Noctort, Queval & Gakière, 2006). Sterols and glycerophospholipids are important constituents of cell membranes and membrane lipids in plants. They play essential roles in membrane asymmetry, diversity, and various physiological and biochemical processes related to plant development and stress response (Du et al., 2022; Vijayakumar et al., 2015). Interestingly, the levels of sterols and glycerophospholipids were up-regulated in waterlogging soil. This indicates that waterlogging may cause damage to cell membranes and membrane lipids, by accumulating sterols and glycerol phospholipids to repair cell membranes and maintain their integrity, thereby enhancing plant resistance.

## Conclusions

Soil amended with pindstrup substrate exhibits a higher water holding capacity, resulting in waterlogging in the soil even after irrigation has ceased in the short term. The waterlogged soil significantly inhibits the growth of roots and stems in *G. sinensis* seedlings. Moreover, it is likely that waterlogging hampers the uptake of Fe and Mn by the roots, impedes the transportation of K from the roots to the stems, and triggers defense mechanisms for Mo absorption. The primary metabolites in thorn grown in waterlogged soil, such as fatty acids and their derivatives, as well as the secondary metabolites, including lignans and phenolic acids, are predominantly suppressed. Hence, when it becomes necessary to acquire these metabolites from the thorns of *G. sinensis*, careful attention must be given to avoid planting trees in high moisture soil and to soil moisture management.

## Supplemental Information

10.7717/peerj.17137/supp-1 Supplemental Information 1 TIC chromatogram of thorn in *G. sinensis* growing in control soil and pindstrup soil. (A) Positive ion mode in control soil; (B) positive ion mode in pindstrup soil; (C) negative ion mode in control soil; (D) negative ion mode in pindstrup.

10.7717/peerj.17137/supp-2 Supplemental Information 2 Supplemental Tables

10.7717/peerj.17137/supp-3 Supplemental Information 3 Raw data

## Abbreviations

UHPLC-MS: ultra-high performance liquid chromatography-mass spectrometry
TN: total nitrogen
TP: total phosphorus
K: potassium
Ca: calcium
Mg: magnesium
Fe: iron
Cu: copper
Zn: zinc
Mo: molybdenum
Mn: manganese
ICP-MS: inductively coupled plasma-mass spectrometry
QC: quality control
SNCE: Sequential Normalized Collision Energy
OPLS-DA: orthogonal projections to latent structures-discriminant analysis
PCA: principal component analysis
VIP: variable importance in the projection
KEGG: Kyoto Encyclopedia of Genes and Genomes
SD: standard deviation
TIC: total ion chromatograms
PM: primary metabolites
SM: secondary metabolites
FCR: ferric-chelate reductase
SOD: superoxide dismutase
TCA: tricarboxylic acid
NADP: nicotinamide adenine dinucleotide phosphate

## Additional Information and Declarations

### Data Availability

The following information was supplied regarding data availability: The raw data is available in the Supplemental File.
