# Supplementary material for: Waterlogging in soil restricts the growth of Gleditsia sinensis seedlings and inhibits the accumulation of lignans and phenolic acids in thorns
Source: PeerJ. 2024 Mar 22;12:e17137. doi: 10.7717/peerj.17137 (PMC10962338; doi:10.7717/peerj.17137)
Supplement: Supplemental Information 1 — (A) Positive ion mode in control soil; (B) positive ion mode in pindstrup soil; (C) negative ion mode in control soil; (D) negative ion mode in pindstrup. [file peerj-12-17137-s001.pdf]

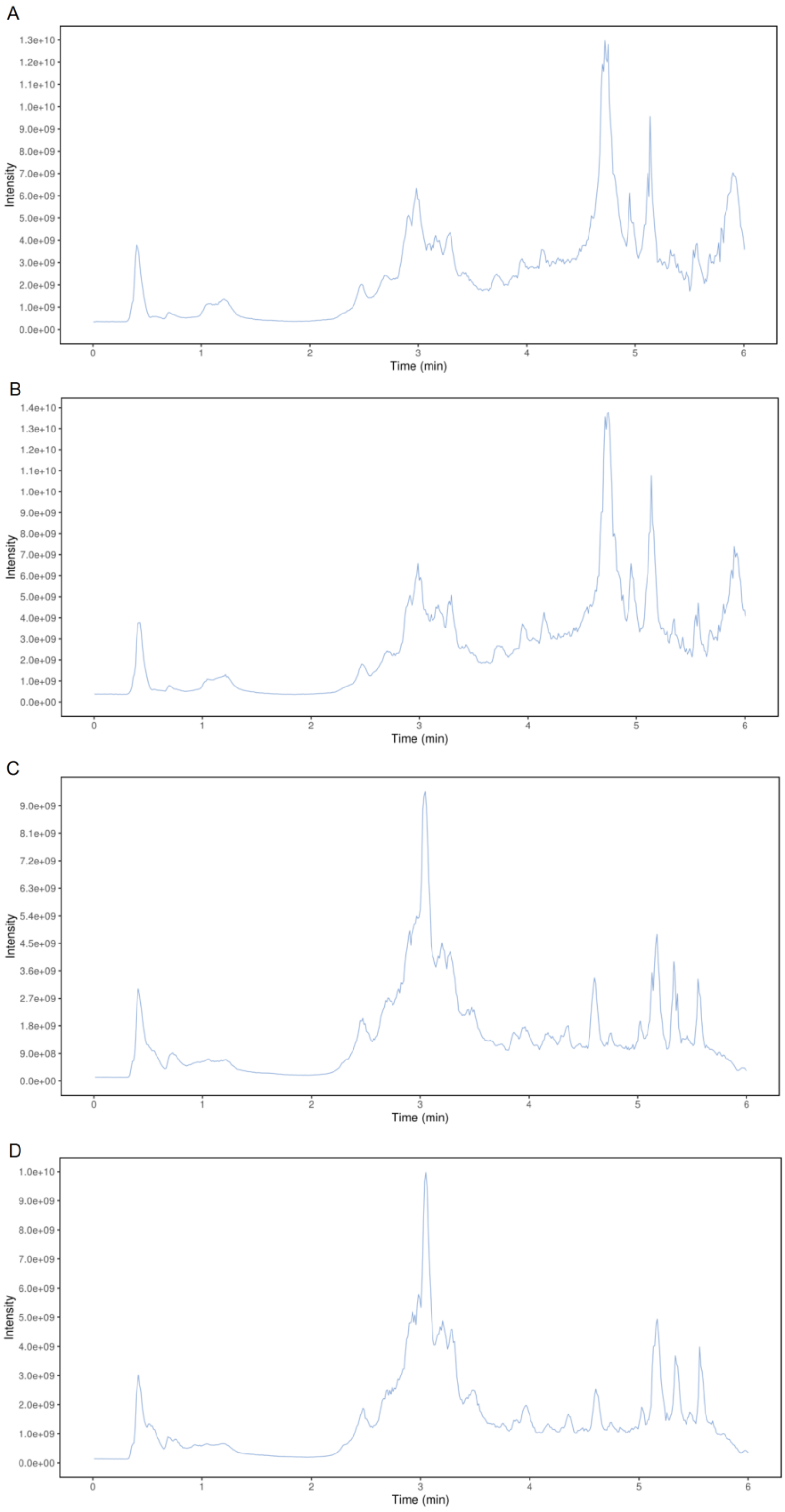

Figure S1. TIC chromatogram of thorn in *G. sinensis* growing in control soil and pindstrup soil. (**A**) positive ion mode in control soil; (**B**) positive ion mode in pindstrup soil; (**C**) negative ion mode in control soil; (**D**) negative ion mode in pindstrup soil.
